# Validation of serrated polyps (SPs) in Swedish pathology registers

**DOI:** 10.1186/s12876-019-1134-6

**Published:** 2019-12-31

**Authors:** Soran R. Bozorg, Mingyang Song, Louise Emilsson, Jonas F. Ludvigsson

**Affiliations:** 1 Department of Medical Epidemiology and Biostatistics, Karolinska Institutet, 171 77 Solna, Sweden; 2 Departments of Epidemiology and Nutrition, Harvard T.H. Chan School of Public Health, Boston, MA USA; 3 Clinical and Translational Epidemiology Unit, Massachusetts General Hospital and Harvard Medical School, Boston, MA USA; 4 Division of Gastroenterology, Massachusetts General Hospital and Harvard Medical School, Boston, MA USA; 5 Institute of Health and Society, University of Oslo, Oslo, Norway; 6 Vårdcentralen Värmlands Nysäter and Centre for Clinical Research, County Council of Värmland, Värmland, Sweden; 7 Department of Paediatrics, Örebro University Hospital, Örebro, Sweden; 8 Division of Epidemiology and Public Health, School of Medicine, University of Nottingham, Nottingham, UK; 9 Department of Medicine, Columbia University College of Physicians and Surgeons, New York, NY USA

**Keywords:** Hyperplastic polyp, Serrated adenoma, Serrated polyp, Sessile serrated adenoma/polyp, Traditional serrated adenoma, Validation

## Abstract

**Background:**

Little is known about the natural history of serrated polyps (SPs), partly due to the lack of large-scale epidemiologic data. In this study, we examined the validity of SP identification according to SNOMED (Systematised Nomenclature of Medicine) codes and free text from colorectal histopathology reports.

**Methods:**

Through the ESPRESSO (Epidemiology Strengthened by histoPathology Reports in Sweden) study, we retrieved data on SPs from all pathology departments in Sweden in 2015–2017 by using SNOMED codes and free-text search in colorectal histopathology reports. Randomly selected individuals with a histopathology report of SPs were validated against patient charts using a structured, retrospective review.

**Results:**

SPs were confirmed in 101/106 individuals with a histopathology report of SPs, yielding a positive predictive value (PPV) of 95% (95%CI = 89–98%). By year of diagnosis, the PPV was 89% (95%CI = 69–97%), 96% (95%CI = 81–99%) and 97% (95%CI = 89–99%) for individuals diagnosed before 2001 (*n* = 19), between 2001 and 2010 (*n* = 26) and after 2010 (*n* = 61), respectively. According to search method, the PPV for individuals identified by SNOMED codes was 100% (95%CI = 93–100%), and 93% (95%CI = 86–97%) using free-text search. Recorded location (colon vs. rectum) was correct in 94% of all SP histopathology reports (95%CI = 84–98%) identified by SNOMED codes. Individuals with SPs were classified into hyperplastic polyps (*n* = 34; 32%), traditional serrated adenomas (n = 3; 3%), sessile serrated adenomas/polyps (SSA/Ps) (*n* = 70; 66%), unspecified SPs (n = 3, 3%), and false positive SPs (*n* = 5, 5%). For individuals identified by SNOMED codes, SSA/Ps were confirmed in 49/52 individuals, resulting in a PPV of 94% (95%CI: 84–98%). In total, 57% had ≥2 polyps (1: *n* = 44, 2–3: *n* = 33 and ≥ 4: *n* = 27). Some 46% of SPs (*n* = 71) originated from the proximal colon and 24% were ≥ 10 mm in size (*n* = 37). Heredity for colorectal cancer, intestinal polyposis syndromes, or both was reported in seven individuals (7%). Common comorbidities included diverticulosis (*n* = 45, 42%), colorectal cancer (*n* = 19, 18%), and inflammatory bowel disease (*n* = 10, 9%).

**Conclusion:**

Colorectal histopathology reports are a reliable data source to identify individuals with SPs.

## Background

Colorectal cancer (CRC) is the third most common cancer and the third leading cause of cancer death worldwide. It kills over 600,000 people annually, accounting for 8% of cancer-related deaths [1]. Adenomatous polyps, now referred to as conventional adenomas, have been regarded as the main precursor of CRC, but in recent years a new pathway to CRC has been identified, termed the serrated pathway [2, 3].

Serrated polyps (SPs) are characterised by a saw-toothed appearance of colonic crypts. As per recommendations from the World Health Organisation (WHO) [4], SPs are classified into three subgroups: hyperplastic polyps (HPs), traditional serrated polyps (TSAs) and sessile serrated adenomas/polyps (SSA/Ps)(Fig. 2 in Appendix). The serrated pathway to CRC is mainly believed to originate from SSA/Ps, which are estimated to represent up to 20% of all SPs [3, 5].

Some data suggest that cancers evolving through the serrated pathway may account for up to 15–30% of all CRC cases, and that they are significantly overrepresented in interval cancers [6], i.e. CRC occurring before the next recommended screening after an initially negative finding. Even though the adenoma-carcinoma pathway still accounts for the majority of the CRC burden, a recent study comparing the risk of CRC development found that the increased risk of CRC in individuals with SPs is similar or higher than that seen in individuals with conventional adenomas [7].

Little is known about the natural history of SP, which may in part be due to the lack of availability of large-scale data. Through the ESPRESSO (Epidemiology Strengthened by histoPathology) study [8], we contacted all pathology departments (*n* = 28) in Sweden to construct a cohort of individuals with an SP diagnosis according to computerised histopathology reports. We then retrieved patient charts from 106 randomly selected individuals with a record of SP. The primary purpose of this study was to validate SP diagnosis according to computerised histopathology reports against patient chart data. A secondary aim was to describe the characteristics of individuals with SPs.

## Methods

We validated SP diagnosis based on computerised histopathology reports in a random subset of individuals through a structured, retrospective review of histopathology reports and patient charts.

### Study population

The ESPRESSO study consists of gastrointestinal histopathology reports from 2.2 million unique individuals with a total of 6.1 million separate data entries. Some 53.9% of individuals had been biopsied more than once. Data on gastrointestinal histopathology reports were collected between October 12, 2015 and April 15, 2017 from all pathology departments in Sweden (*n* = 28). Overall we had data on 1,618,953 colon biopsies and 771,511 rectal biopsies [8]. Through the unique personal identity number [9] assigned to all Swedish residents, histopathology data were linked to the Swedish national health registers (Patient Register [10], Cause of Death Register [11], Cancer Register [12], Medical Birth Register [13], Prescribed Drug Register [14], The LISA database with socioeconomic data [15], as well as the Total Population Register [16]). Details about ESPRESSO and registry linkage have been described previously [8].

For the current study on SPs, we included individuals with a colorectal biopsy (topography codes: T67–68) with the following Systematised Nomenclature of Medicine (SNOMED) codes: M82160, M8216, M82130, M8213. We also included individuals with a colorectal biopsy of which the histopathology report free text listed “serrated polyp” (Swedish “sågtand(ad)”).

### Study sample

**Fig. 1:**
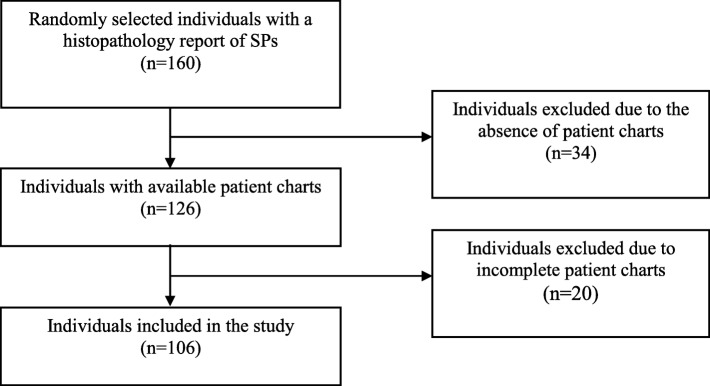
Flowchart for inclusion of individuals in the study. (Abbreviations: SPs = Serrated polyps)

**Fig. 2:**
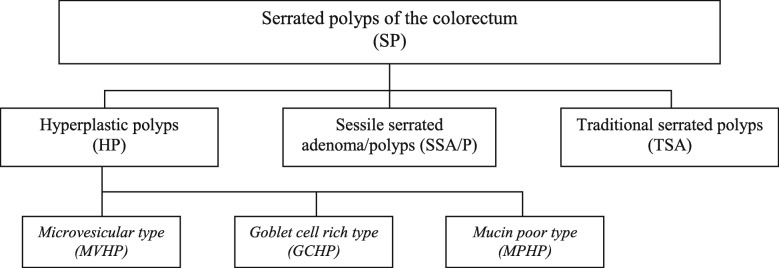
Nomenclature of serrated polyps of the colorectum according to the World Health Organisation (WHO) taxonomy [4]

Power calculation using EpiTools [17] indicated a minimum of 139 individuals were needed to obtain a positive predictive value (PPV) for SP of 90% with a 95% confidence interval (95%CI) range of 85–95% (using an alpha of 0.05 and a beta of 0.20). For this validation, we requested patient charts from a random sample of 160 individuals with a histopathology report of SPs from five Swedish counties. We were able to retrieve patient chart data from 126 individuals, out of which 106 had sufficient information for our validation (Fig. 1).

### Case definition

We defined a true SP as having a consistent histopathology report and a patient chart supporting an SP diagnosis. Individuals with an SP diagnosis could have one or multiple SPs. Assessment of histopathology reports and patient charts was executed by the principal author (SRB). Uncertain cases were discussed with JFL and MS. If no consensus was reached, the case was considered inconsistent with SP.

### Data elements

Data from patient charts were extracted using a standardised form, similar to the form used by *Svensson* et al. in their validation of microscopic colitis [18]. The starting point of data extraction was set to 2 years before the diagnosis until March 2018. The data from the patient charts mainly included patient history, laboratory data, referral letters and endoscopy and histopathology reports. Individuals were excluded in the absence of a histopathology report or insufficient/incomplete data.

### Statistics

The main outcome of this study was the PPV for SP diagnosis in the 106 individuals with patient charts containing sufficient data. To identify any potential differences, results were stratified according to search method (SNOMED codes or free-text search). Given the changing nomenclature of SPs over time, we also analysed the data by year of diagnosis. For individuals identified by SNOMED codes, we validated the SP location by comparing the topography code with the patient chart. SNOMED codes were also used to identify SSA/Ps, for which a separate PPV was calculated. We estimated 95%CIs with the Wilson score interval [19] using EpiTools [20].

In addition to retrieving colonoscopy and histopathology reports, we collected data on sex, age, year of diagnosis, smoking, obesity, comorbidity, diagnostic tools and indication for endoscopy. For evaluation of anaemia, we used 132 g/L for men and 122 g/L for women as the lower limits of normal haemoglobin concentration as proposed by Beutler and Waalen [21]. The size of polyp characteristics was determined as either larger or smaller than 10 mm as this size has been proposed as the threshold for determining the future management of SPs [22]. Other aspects investigated were number of polyps (0, 1, 2–3 or ≥ 4), location (proximal, distal, rectal) and grade of dysplasia (none, low, high). The proximal colon was defined as the ileocecal valve until the splenic flexure, followed by the distal colon until the last 10 cm of the gastrointestinal tract that represent the rectum.

For the descriptive analysis, we calculated the population and polyp characteristics according to SP subgroups. To reflect the previous version of the WHO recommendations on SP classification, SPs described as serrated adenomas (SAs) or mixed polyps with a serrated component were deemed consistent with SSA/P [23]. Nonetheless, data were also analysed separately for these polyp subgroups. Data on false positive SPs were also presented separately.

## Results

### Data

The charts of 106 individuals were retrieved from pathology centres distributed in five counties in Sweden: Dalarna, Norrbotten, Skaraborg, Stockholm and Örebro.

### Positive predictive value (PPV)

SPs were confirmed in 101/106 individuals, yielding a PPV of 95% (95%CI = 89–98%) (Table 1). Of the five individuals with false positive SPs, one had SP ruled out by the pathologist. The other four individuals had SPs mentioned in the histopathology report but sufficient evidence to confirm the diagnosis was lacking. No false positive case was found among individuals identified by SNOMED codes (*n* = 52), resulting in a PPV of 100% (95%CI = 93–100%). For individuals identified by free-text search of histopathology reports (*n* = 76), the PPV was 93% (95%CI = 86–97). Out of these, 22 individuals also had a SNOMED code.

**Table 1:** Positive predictive value of serrated polyps in Swedish histopathology reports

Variable	SNOMED codes M8213(0)/M8216(0)	Free text “serrated”	Total
Individuals	52 (49%)	76 (72%)	106 (100%)
By subtype			
*Hyperplastic polyp*	5 (10%)	31 (41%)	34 (32%)
*Traditional serrated adenoma*	2 (4%)	3 (4%)	3 (3%)
*Sessile serrated adenoma/polyp*	49 (94%)	40 (53%)	70 (66%)
*Unspecified serrated polyp*	0 (0%)	3 (4%)	3 (3%)
*False positive serrated polyp*	0 (0%)	5 (7%)	5 (5%)
PPV, SP diagnosis	52/52, 100% (95%CI = 93–100%)	71/76, 93% (95%CI = 86–97%)	101/106, 95% (95%CI = 89–98%)
By year of diagnosis			
*< 2000*	0/0	17/19, 89%	17/19, 89%
*2000–2009*	16/16, 100%	10/11, 91%	25/26, 96%
*≥ 2010*	36/36, 100%	44/46, 96%	59/61, 97%
PPV, location	49/52, 94% (95%CI = 84–98%)	–	–
By topography code			
*T67*	25/25, 100%	–	–
*T671*	1/1, 100%	–	–
*T672*	3/3, 100%	–	–
*T677*	2/2, 100%	–	–
*T68*	18/21, 86%	–	–
PPV, SSA/P diagnosis	49/52, 94% (95%CI = 84–98%)	–	–

Abbreviations: CI = Confidence interval; PPV = Positive predictive value; SP = Serrated polyp; SNOMED = Systematised Nomenclature of Medicine

By year of diagnosis, the PPV was 89% (95%CI = 69–97%), 96% (95%CI = 81–99%) and 97% (95%CI = 89–99%) for individuals diagnosed before 2001 (*n* = 19), between 2001 and 2010 (*n* = 26) and after 2010 (*n* = 61), respectively. All individuals diagnosed before 2000 were identified by free-text entries alone, while 69% (*n* = 36) of the individuals identified by SNOMED codes were diagnosed after 2010. For individuals identified by SNOMED codes, the recorded location was accurate in 49/52 (94%, 95%CI = 84–98%) cases of all SP histopathology reports. The three individuals with an incorrect recorded topography code had been biopsied in the distal (sigmoid) colon but recorded as having their polyp in the rectum (T68). Only five individuals had a subsite-specific topography code within the colon (T671-T677), all of which were accurate.

Most individuals with SSA/Ps were identified by SNOMED codes (*n* = 49, 70%), whereas most individuals with HPs were identified by free-text searches (*n* = 31, 91%). Of all individuals identified by SNOMED codes, SSA/Ps were confirmed in 49/52 individuals, resulting in a PPV of 94% (95%CI = 84–98%). The false positive cases consisted of two TSAs (*n* = 2) and one HP (*n* = 1).

### Demographics and risk factors

Of the 106 validated individuals, 50 were female (47%) and the median age at diagnosis was 70 years (Table 2). Most SP cases were diagnosed through colonoscopy (*n* = 86, 81%), with smaller proportions diagnosed through partial lower endoscopy (sigmoidoscopy, rectoscopy or proctoscopy, *n* = 15, 14%) or hemicolectomy (*n* = 5, 5%). The data were stratified as follows: HP (*n* = 34, 32%), TSA (*n* = 3, 3%), SSA/P (*n* = 70, 66%), unspecified SP (*n* = 3, 3%), and false positive SP (*n* = 5, 5%). The SSA/P subgroup also included polyps described as serrated adenomas (*n* = 51) and mixed polyps (*n* = 12). Because some individuals had polyps of different subtypes (*n* = 9), the sum of individuals in the subgroups exceeds the total number of individuals reviewed. Notably, the HP subgroup was diagnosed earlier than the SPs overall (median year: 2003 vs. 2012) and there were no polyps specifically described as an SSA/P or TSA before 2011. Polyps described specifically as serrated adenomas were reported as early as 2002. Otherwise, population characteristics were similar in the different SP subgroups.

**Table 2:** Demographics of individuals with serrated polyps according to Swedish histopathology reports

Variable	HP	TSA	SSA/P				U	F	Total
			SSA/P	SA	Mixed	Total			
INDIVIDUALS	34 (32%)	3 (3%)	8 (8%)	51 (48%)	12 (11%)	70 (66%)	3 (3%)	5 (5%)	106 (100%)
Year of diagnosis									
*Min*	1989	2015	2011	2002	2008	2002	2005	1991	1989
*Median*	2003	2015	2014	2013	2014	2014	2008	2007	2012
*Max*	2016	2016	2016	2015	2015	2016	2014	2014	2016
Age									
*Min*	39	52	59	41	53	41	62	35	35
*Median*	68	58	64	74	74	74	65	73	70
*Max*	82	66	78	93	85	93	70	87	93
Sex									
*Female*	15 (44%)	0 (0%)	5 (63%)	26 (51%)	5 (42%)	35 (50%)	3 (100%)	1 (20%)	50 (47%)
Detection procedure									
*Colonoscopy*	28 (82%)	2 (67%)	7 (88%)	40 (78%)	10 (83%)	57 (87%)	3 (100%)	5 (100%)	86 (81%)
Partial lower endoscopy*	5 (15%)	1 (33%)	0 (0%)	9 (18%)	0 (0%)	9 (0%)	0 (0%)	0 (0%)	15 (14%)
*Colectomy*	1 (3%)	0 (0%)	1 (13%)	2 (4%)	2 (17%)	4 (13%)	0 (0%)	0 (0%)	5 (5%)

Abbreviations: F = False positive serrated polyp; HP = Hyperplastic polyp; SA = Serrated adenoma; SSA/P = Sessile serrated adenoma/polyp; TSA = Traditional serrated adenoma; U = Unspecified serrated polyp. *Sigmoidoscopy, rectoscopy or proctoscopy

At diagnosis, 16 (15%) individuals were current smokers, whereas 14 (13%) had a record of earlier smoking (Table 3). Obesity (body mass index, BMI ≥30 or indication of obesity in the patient chart) was seen in 12 individuals (11%). Heredity for CRC, intestinal polyposis syndromes, or both was reported in seven individuals (7%). Common comorbidities consisted of diverticulosis (*n* = 45, 42%), conventional adenomas (*n* = 33, 31%), CRC (*n* = 19, 18%) and inflammatory bowel disease (IBD) (*n* = 10, 9%). Comorbidities were defined as having a diagnosis prior to or in conjunction with a diagnosis of SP, except for conventional adenomas for which prior diagnoses were not considered.

**Table 3:** Risk factors and comorbidity of individuals with serrated polyps according to Swedish histopathology reports

Variable	HP	TSA	SSA/P				U	F	Total
			SSA/P	SA	Mixed	Total			
INDIVIDUALS	34 (32%)	3 (3%)	8 (8%)	51 (48%)	12 (11%)	70 (66%)	3 (3%)	5 (5%)	106 (100%)
Smoking									
*Current*	8 (24%)	1 (33%)	1 (13%)	2 (4%)	4 (33%)	7 (10%)	2 (67%)	0 (0%)	16 (15%)
*Previous*	5 (15%)	2 (67%)	3 (38%)	5 (10%)	2 (17%)	10 (14%)	0 (0%)	0 (0%)	14 (13%)
BMI									
Obese*	3 (9%)	2 (67%)	2 (25%)	5 (10%)	1 (8%)	8 (11%)	1 (33%)	0 (0%)	12 (11%)
Heredity									
*CRC +* ^*§*^	2 (6%)	0 (0%)	2 (25%)	1 (2%)	1 (8%)	4 (6%)	1 (33%)	0 (0%)	7 (7%)
Comorbidity									
*Diverticulosis*	9 (26%)	1 (33%)	4 (50%)	24 (47%)	8 (67%)	36 (51%)	1 (33%)	1 (20%)	45 (42%)
*CRC*	3 (9%)	1 (33%)	3 (38%)	10 (20%)	3 (25%)	15 (21%)	2 (67%)	0 (0%	19 (18%)
*IBD*	4 (12%)	0 (0%)	0 (0%)	5 (10%)	1 (8%)	6 (9%)	0 (0%)	0 (0%)	10 (9%)

Abbreviations: BMI = Body mass index; CRC = Colorectal cancer; F = False positive serrated polyp; HP = Hyperplastic polyp; IBD = Inflammatory bowel disease; SA = Serrated adenoma; SSA/P = Sessile serrated adenoma/polyp; TSA = Traditional serrated adenoma; U = Unspecified serrated polyp. *BMI ≥ 30 or indication of obesity in patient chart, ^*§*^*CRC and/or intestinal polyposis syndromes*

### Indications and symptoms

Most individuals underwent endoscopy for clinical symptoms (*n* = 64, 60%) (Table 4). For individuals with symptomatic indications and SP as their only significant endoscopic finding (*n* = 28), the most frequent symptoms were change in stool form (diarrhoea or obstipation, *n* = 21, 75%), change in stool colour (haematochezia or melena, *n* = 14, 50%) and anaemia (*n* = 11, 39%). Endoscopies carried out due to an asymptomatic indication (*n* = 39, 37%) mostly consisted of surveillance endoscopies due to a history of previous polyps or adenomas (*n* = 18, 46%), a history of CRC (n = 3, 8%) or a history of IBD (*n* = 5, 13%). CRC screening was also a frequent indication of asymptomatic endoscopies (*n* = 8, 21%), out of which six individuals also had a positive faecal occult blood test (FOBT) prior to the endoscopy.

**Table 4:** Endoscopy indication and symptoms of individuals with serrated polyps according to Swedish histopathology reports

Variable	HP	TSA	SSA/P				U	F	Total
			SSA/P	SA	Mixed	Total			
INDIVIDUALS	34 (32%)	3 (3%)	8 (8%)	51 (48%)	12 (11%)	70 (66%)	3 (3%)	5 (5%)	106 (100%)
Indication									
*Symptomatic*	18 (53%)	1 (33%)	3 (38%)	35 (69%)	8 (67%)	45 (64%)	1 (33%)	4 (80%)	64 (60%)
Non-symptomatic*	15 (44%)	2 (67%)	5 (63%)	14 (27%)	4 (33%)	23 (33%)	2 (67%)	1 (20%)	39 (37%)
*Unspecified*	1 (3%)	0 (0%)	0 (0%)	2 (4%)	0 (0%)	2 (3%)	0 (0%)	0 (0%)	3 (3%)
Symptoms									
*Change in stool form*	16 (47%)	1 (33%)	2 (25%)	23 (45%)	5 (42%)	30 (43%)	0 (0%)	2 (40%)	47 (44%)
*Change in stool colour*	9 (26%)	0 (0%)	3 (38%)	22 (43%)	3 (25%)	27 (39%)	1 (33%)	1 (20%)	36 (34%)
*Abdominal pain*	5 (15%)	0 (0%)	4 (50%)	10 (20%)	2 (17%)	15 (21%)	1 (33%)	1 (20%)	22 (21%)
*Weight loss*	2 (6%)	0 (0%)	0 (0%)	6 (12%)	1 (8%)	7 (10%)	1 (33%)	0 (0%)	9 (8%)
*Fatigue*	2 (6%)	0 (0%)	0 (0%)	4 (8%)	2 (17%)	6 (9%)	0 (0%)	0 (0%)	8 (8%)
*Anaemia*	6 (18%)	0 (0%)	1 (13%)	18 (35%)	4 (33%)	22 (31%)	1 (33%)	2 (40%)	30 (28%)
*Positive FOBT*	4 (12%)	3 (100%)	1 (13%)	4 (8%)	3 (25%)	8 (11%)	2 (67%)	0 (0%)	15 (14%)
*Others*^*§*^	4 (12%)	0 (0%)	0 (0%)	5 (10%)	0 (0%)	5 (7%)	0 (0%)	0 (0%)	8 (8%)

Abbreviations: F = False positive serrated polyp; FOBT = Faecal occult blood test; HP = Hyperplastic polyp; SA = Serrated adenoma; SSA/P = Sessile serrated adenoma/polyp; TSA = Traditional serrated adenoma; U = Unspecified serrated polyp. *Polyp surveillance, CRC screening, etc.*,* ^*§*^*Fever, burning, nausea,* etc.

The most frequent symptoms in individuals in our cohort, regardless of endoscopy indication, were change in stool form (*n* = 47, 44%), change in stool colour (*n* = 36, 34%), anaemia (*n* = 30, 28%), abdominal pain (*n* = 22, 21%), weight loss (*n* = 9, 8%) and fatigue (n = 8, 8%). Other less frequent symptoms included nausea (n = 4, 4%), anal burning (n = 2, 2%), fever (*n* = 1, 1%), loss of appetite (n = 1, 1%) and dyspnoea (n = 1, 1%). Fifteen individuals (14%) had a positive FOBT before endoscopy. In total, clinical signs of gastrointestinal bleeding (change in stool colour, anaemia or FOBT) were seen in 58 (55%) individuals in total and in 28 (61%) individuals with SP as their only endoscopic finding.

### Polyp characteristics

According to endoscopy reports, 44 (42%) individuals had one polyp at diagnosis, 33 (31%) had 2–3 polyps and 27 (25%) had ≥4 polyps (Table 5). Two persons with a false positive SP had no certain polyps. The total number of polyps was 155, which could be classified into HPs (*n* = 61, 39%), TSAs (*n* = 3, 2%), SSA/Ps (*n* = 80, 52%), unspecified SPs (n = 8, 5%) and false positive SPs (n = 3, 2%). The size of the polyps was determined as either large (≥10 mm) or small (< 10 mm). In all, there were 58 (37%) small polyps and 37 (24%) large polyps. Only four (7%) HPs were considered large, out of which two were proximal. In contrast, all TSAs were large (n = 3, 100%), whereas SSA/Ps presented a more even distribution regarding size (small: *n* = 31, 39%, large: *n* = 28, 35%). In terms of location unspecified SPs were predominantly found in the proximal colon (*n* = 7, 88%), whereas SSA/Ps were generally found either proximally (*n* = 39, 49%) or rectally (*n* = 26, 33%). TSAs were seen in the distal colon (n = 1, 33%) or rectum (n = 2, 67%), whereas HPs were relatively evenly distributed.

**Table 5:** Characteristics of serrated polyps

Variable	HP	TSA	SSA/P				U	F	Total
			SSA/P	SA	Mixed	Total			
Individuals	34 (32%)	3 (3%)	8 (3%)	51 (48%)	12 (11%)	70 (66%)	3 (3%)	5 (5%)	106 (100%)
Number of polyps*									
*0*	0 (0%)	0 (0%)	0 (0%)	0 (0%)	0 (0%)	0 (0%)	0 (0%)	2 (40%)	2 (2%)
*1*	8 (24%)	2 (67%)	4 (50%)	24 (47%)	5 (42%)	33 (47%)	0 (0%)	1 (20%)	44 (42%)
*2–3*	11 (32%)	1 (33%)	2 (25%)	15 (29%)	4 (33%)	21 (30%)	1 (33%)	2 (40%)	33 (31%)
*≥ 4*	15 (44%)	0 (0%)	2 (25%)	12 (24%)	3 (25%)	16 (23%)	2 (67%)	0 (0%)	27 (25%)
Simultaneous findings									
*Conventional adenoma*	15 (44%)	0 (0%)	0 (0%)	17 (33%)	5 (42%)	22 (31%)	0 (0%)	1 (20%)	33 (31%)
*CRC*	1 (3%)	0 (0%)	3 (38%)	5 (10%)	1 (8%)	8 (11%)	1 (33%)	0 (0%)	10 (9%)
POLYPS	61 (39%)	3 (2%)	11 (7%)	56 (36%)	13 (8%)	80 (52%)	8 (5%)	3 (2%)	155 (100%)
Size									
*< 10*	26 (43%)	0 (0%)	5 (45%)	24 (43%)	2 (15%)	31 (39%)	0 (0%)	1 (33%)	58 (37%)
*≥ 10*	4 (7%)	3 (100%)	5 (45%)	16 (29%)	7 (54%)	28 (35%)	2 (25%)	0 (0%)	37 (24%)
*Unspecified*	31 (51%)	0 (0%)	1 (9%)	16 (29%)	4 (31%)	21 (26%)	6 (75%)	2 (67%)	60 (39%)
Location									
*Proximal*	25 (41%)	0 (0%)	9 (82%)	23 (41%)	7 (54%)	39 (49%)	7 (88%)	0 (0%)	71 (46%)
*Distal*	25 (41%)	1 (33%)	2 (18%)	8 (14%)	5 (38%)	15 (19%)	1 (13%)	2 (67%)	44 (28%)
*Rectal*	11 (18%)	2 (67%)	0 (0%)	25 (45%)	1 (8%)	26 (33%)	0 (%)	1 (33%)	40 (26%)
*Unspecified*	0 (0%)	0 (0%)	0 (0%)	0 (0%)	0 (0%)	0 (0%)	0 (0%)	0 (0%)	0 (0%)
Dysplasia									
*None*	16 (26%)	0 (0%)	2 (18%)	3 (5%)	0 (0%)	5 (6%)	5 (63%)	0 (0%)	26 (17%)
*Low*	2 (3%)	3 (100%)	4 (36%)	45 (80%)	12 (92%)	61 (76%)	1 (13%)	3 (100%)	70 (45%)
*High*	0 (0%)	0 (0%)	1 (9%)	3 (5%)	1 (8%)	5 (6%)	0 (0%)	0 (0%)	5 (3%)
*Unspecified*	43 (70%)	0 (0%)	4 (36%)	5 (9%)	0 (0%)	9 (11%)	2 (25%)	0 (0%)	54 (35%)

Abbreviations: CRC = Colorectal cancer; F = False positive serrated polyp; HP = Hyperplastic polyp; SA = Serrated adenoma; SSA/P = Sessile serrated adenoma/polyp; TSA = Traditional serrated adenoma; U = Unspecified serrated polyp. *Includes conventional adenomas

Evaluating the grade of dysplasia, 26 polyps had no sign of dysplasia (17%). Low-grade dysplasia was seen in 70 (45%) polyps and high grade in 5 (3%). Polyps with no dysplasia were overrepresented among HPs (*n* = 16, 26%) and unspecified SPs (*n* = 5, 63%). In 43 (70%) HPs degree of dysplasia was not specified. The number of polyps with unspecified degree of dysplasia in the other subgroups was 0 (0%) for TSA, 9 (11%) for SSA/P and 2 (25%) for unspecified SP. Most TSAs and SSA/Ps exhibited low-grade dysplasia (TSA: *n* = 3, 100%; SSA/P: *n* = 61, 76%); cases of high-grade dysplasia were only seen in SSA/Ps (n = 5, 6%).

## Discussion

Our study found a high PPV (95%, 95%CI: 89–98%) for SPs according to colorectal histopathology reports based on SNOMED codes and free-text searches. The high PPV was similar over time. This finding suggests that histopathology reports are a reliable source to identify individuals with SPs. The PPV of this study is comparable with that of other gastrointestinal diagnoses based on histopathology: celiac disease (PPV 95%) and microscopic colitis (PPV 95%) [18, 24]. The high specificity for SPs is not surprising given that the assignment of the SNOMED code and free-text diagnosis is already based on histopathological evaluation.

As to search method, the use of SNOMED codes to identify individuals with SPs had a higher specificity than the use of free-text search (PPV: 100% vs. 93%), but still the PPV using free text is consistent with the accuracy of having a physician-assigned diagnosis in the Swedish Patient Register (95%CI PPV = 85–95%) [10]. Furthermore, the high PPV of SSA/P among individuals identified through SNOMED codes (94%, 95%CI: 84–98%) indicates that an exclusive use of SNOMED codes can serve to target these polyps specifically. For individuals identified by SNOMED codes, the corresponding topography codes can also be used to determine the location of the SPs and SSA/Ps (PPV: 94%; 95%CI = 84–98%). The cases of incorrect topography codes exclusively concerned individuals with a rectal topography code (T68), which were classified as distal (sigmoidal) according to our validation. This discrepancy occurred because we mainly used endoscopy reports to determine the macroscopic location of the polyps, whereas topography codes are assigned by the pathologist and sometimes based on histological appearance.

Subsequent to the recognition of the different SP subgroups, several studies have investigated their respective prevalence. HPs have consistently been shown to be the most common subtype, representing 70–90% of all SPs [25–27]. Likewise, SSA/Ps have been shown to represent up to 10–25% of all SPs while TSAs represent about 1% [25–29]. In our study, we primarily targeted SSA/Ps. As such, we did not include SNOMED codes for HPs. Consequently, the proportion of HPs in our cohort does not reflect the overall proportion among SPs, as HPs are likely to have been included when they have been described as “serrated” in the histopathology report. As a result, most individuals with HPs have been identified by free-text searches (*n* = 31, 91%).

Given the evolving nomenclature of SPs, a large number of polyps in our study were described following the previous version of the WHO classification of colorectal polyps published in 2000 [23]. This version recognised HPs separately and SAs as a subtype under adenomas. Within the SA subtype, there was no differentiation between SSA/Ps and TSAs. As such, polyps described as serrated adenomas can represent any of these two. However, given the predominate prevalence of SSA/Ps, it is reasonable to assume that the number of TSAs described as serrated adenomas is small. It is also reassuring to note that the specific SP descriptions correlated well with the publication year of the different WHO classifications, i.e. polyps described as serrated adenomas began to appear after 2000 and polyps described as SSA/Ps or TSAs were found only after 2010.

The individuals in our study were equally distributed in terms of sex (female: 47%). However, the mean age of the cohort was 70 (range: 35–93) years, which is slightly higher than that found in previous studies [3, 26, 28, 30]. The age difference may, to some extent, be explained by the high proportion of dysplastic SPs in our study (*n* = 75, 48%). Heredity, smoking and obesity have all been established as risk factors for SP, with smoking being more strongly linked to SSA/Ps than to the other subgroups [31–34]. In this study mention of risk factors in the patient chart was regarded as indicative of that risk factor, while, for instance, an individual in which smoking was not mentioned in the patient chart was regarded as a non-smoker. Thus, the prevalence of some risk factors may have been underestimated. For instance, only 11% of our individuals had a record of obesity compared with 16% in the general Swedish population despite evidence showing that obesity is a risk factor for SP [31, 32, 35].

Several studies have established low detection as a significant challenge in SP research, and endoscopy screening seems less effective for detecting proximal CRC, which is believed to originate predominantly from the serrated pathway [6, 25, 36–39]. Moreover, HPs are considered less likely to bleed compared with adenomas, and SSA/Ps lack some genetic markers currently used in DNA faecal tests, decreasing the sensitivity of faecal tests for SPs.

In our study 15 (14%) individuals had a positive FOBT prior to endoscopy and 58 (55%) unique individuals had at least one sign of gastrointestinal bleeding (FOBT, haematochezia/melena or anaemia). To some extent the high percentage of individuals with SPs and signs of gastrointestinal bleeding can be explained by the simultaneous presence of adenomas (*n* = 18, 31%), as well as the overrepresentation of SPs other than HPs. However, we cannot exclude that bleeding-prone SPs are overrepresented in our cohort.

Most individuals with SPs underwent endoscopy due to clinical symptoms (*n* = 64, 60%). In addition, regardless of endoscopy indication, we found that 78 individuals (74%) had at least one symptom (which includes positive FOBT) at the time of diagnosis, including one individual with a false positive SP. Of note, false positive cases more often presented with clinical symptoms as an indication for endoscopy (80% vs. 60%).

In a notable proportion of HPs (*n* = 43, 73%), grade of dysplasia was not specified. The reason for this is probably that HPs are normally defined as non-dysplastic. Thus, any specification of dysplasia by the pathologist would therefore be redundant considering that it is already implied by the HP diagnosis [40]. As such, the proportion of HPs without dysplasia should be interpreted as 97% (59/61) instead of 26% (16/61). Among the polyps classified as SAs, the vast majority exhibited low-grade dysplasia (*n* = 45, 80%) and there were only three polyps (5%) with no dysplasia. This observation reinforces the idea that polyps described as SAs are consistent with SSA/Ps, or possibly TSAs, as HPs are typically non-dysplastic [40]. More specifically, consistent with the literature on SSA/P location, we believe that proximal SAs will almost exclusively consist of SSA/Ps. However, SAs located in the rectum are likely to include a small number of TSAs.

The literature has shown that only about 15% of SSA/Ps have any dysplastic features, implying that SSA/Ps with dysplasia are overrepresented in our study [28]. We cannot rule out that a few SSA/Ps with no dysplasia may have been misclassified as HPs given the established difficulty of distinguishing SSA/Ps from large proximal HPs [41]. Yet, it is also possible that SSA/Ps without dysplasia may have been overlooked and left undetected to a larger extent than SSA/Ps with dysplasia.

### Strengths and limitations

The main strength of our study is the random selection of individuals with SPs from a nationwide histopathology cohort. Using a standardised form, we were able to examine not only the PPV for a histopathology report with SP but also describe Swedish individuals with SPs for clinical characteristics and risk factors. Our results are consistent with similar studies for which the gold standard of diagnosis is biopsy, further reinforcing the reliability of the present results.

A limitation of our study includes the lack of re-examinations of actual biopsies. The ethics review board allowed us to collect digital data but not actual tissue samples. Instead, the validation was based on re-evaluation of patient charts that included, among other things, histopathology and endoscopy reports. The quality of the patient chart data varied, especially in the documentation of risk factors and symptoms. Still, given that SP is a strictly histopathological diagnosis, the difference in data availability among the individuals should not have affected the validation in that all individuals had to have the corresponding histopathology report available to be included in the study.

Earlier studies have shown inter-observer variability for classification of SPs among pathologists [42, 43], and we cannot rule out some misclassification, especially for the subgroup classification. This could potentially affect the validity of SSA/P since some of the SSA/Ps may have been misdiagnosed as HPs, and vice versa [43]. The diversity of pathologists in this study, where some may not specialize in SPs, may have decreased the accuracy in polyp classification.

## Conclusion

In conclusion, this study suggests that colorectal histopathology reports are a reliable data source to identify individuals with SPs.

## Data Availability

The datasets used and/or analysed during the current study are available from JFL on reasonable request.

## Abbreviations

CI: Confidence interval
CRC: Colorectal cancer
FOBT: Faecal occult blood test
HP: Hyperplastic polyp
IBD: Inflammatory bowel disease
PPV: Positive predictive value
SNOMED: Systematised nomenclature of medicine
SP: Serrated polyp
SSA/P: Sessile serrated adenoma/polyp
TSA: Traditional serrated adenoma
WHO: World Health Organisation
